# *Special Issue*: Sleep Bruxism—The Controversial Sleep Movement Activity

**DOI:** 10.3390/jcm9030880

**Published:** 2020-03-23

**Authors:** Mieszko Wieckiewicz, Efraim Winocur

**Affiliations:** 1 Department of Experimental Dentistry, Wroclaw Medical University, 50425 Wroclaw, 26 Krakowska St., Poland; 2 Department of Oral Rehabilitation, Faculty of Medicine, The Maurice and Gabriela Goldschleger School of Dental Medicine, Tel Aviv University, P.O.B 39040, Ramat Aviv, Tel Aviv 69978, Israel; winocur@tauex.tau.ac.il

According to the current approach [1], bruxism is considered as two different behaviours observed during sleep and wakefulness, respectively, and the single definition for bruxism has been replaced by two separate definitions:

1. Sleep bruxism is a masticatory muscle activity during sleep that is characterised as rhythmic (phasic) or non-rhythmic (tonic) and is not a movement disorder or a sleep disorder in otherwise healthy individuals.
2. Awake bruxism is a masticatory muscle activity during wakefulness that is characterised by repetitive or sustained tooth contact and/or by bracing or thrusting of the mandible and is not a movement disorder in otherwise healthy individuals.

The prevalence of sleep bruxism among children and adolescents is 3%–49% [2]. Even though the etiopathogenesis of sleep bruxism is not fully understood [3], many different factors are believed to be associated with this muscular activity [4]. An increasing number of evidences suggest a relationship between sleep bruxism and other disorders or systemic diseases, including sleep breathing disorders, uncontrolled limb movements during sleep, reflux disease, and neurological disorders [5].

This *Special Issue* aims to determine the nature of sleep bruxism and its types, as well as to investigate the relationship between sleep bruxism and different general diseases, health conditions, and disorders.

The reader could find in this *Special Issue* a lot of current and useful information about:

1. The co-occurrence of sexsomnia, sleep bruxism, and other sleep disorders.
2. Evaluation of biofeedback usefulness in masticatory muscle activity management.
3. Evaluation of the intensity of sleep bruxism in arterial hypertension.
4. Associations among bruxism, gastroesophageal reflux disease, and tooth wear.
5. *Origanum majorana* essential oil inhalation during neurofeedback training reduces saliva myeloperoxidase activity at session 1 in bruxistic patients.
6. The prevalence of function-dependent temporomandibular joint and masticatory muscle pain and predictors of temporomandibular disorders among patients with Lyme disease.
7. The Bruxoff device as a screening method for sleep bruxism in dental practice.
8. The correlation between sleep bruxism, stress, and depression.
9. The relationship between sleep bruxism and obstructive sleep apnoea.
10. The myorelaxant effect of transdermal cannabidiol application in patients with temporomandibular disorders (TMD).
11. The relationship between sleep bruxism intensity and renalase concentration.
12. Pain predictors in a population of temporomandibular disorder patients.
13. Correlations between sleep bruxism and temporomandibular disorders.

We strongly encourage readers to take a look at the articles that have been published in this *Special Issue*.
